# Lost opportunities for mismatch repair (MMR) screening among minority women with endometrial cancer

**DOI:** 10.1038/s41598-021-91053-1

**Published:** 2021-06-03

**Authors:** Marilyn Huang, Tegan Hunter, Lydia A. Fein, Johnny Galli, Sophia George, Matthew Schlumbrecht, Kelly McCarter, Abdulrahman K. Sinno, Luiz P. Guido, Andre Pinto

**Affiliations:** 1 Division of Gynecologic Oncology, Sylvester Comprehensive Cancer Center, University of Miami Miller School of Medicine, 1121 NW 14th St, Suite 345E, Miami, FL 33136 USA; 2 University of Miami Miller School of Medicine, 1600 NW 10th Avenue, Miami, FL 33136 USA; 3 Department of Obstetrics, Gynecology and Reproductive Medicine, University of Miami Miller School of Medicine, 1120 NW 14th Street, Miami, FL 33136 USA; 4 Department of Pathology, University of Miami Miller School of Medicine, 1120 NW 14th Street, Miami, FL 33136 USA

**Keywords:** Cancer prevention, Endometrial cancer

## Abstract

Lynch Syndrome (LS) prevalence in underrepresented minorities are lacking. The objective of this study was to assess the prevalence of LS in a minority patient population. Secondary objectives included identifying factors associated with successful LS screening and to characterize clinicopathologic features. Women with endometrial cancer treated within a university system from 2014 and 2016 were included. Immunohistochemistry (IHC) results of MLH1, PMS2, MSH2 and MSH6 were obtained from medical records and clinicopathologic factors abstracted. Patients not previously screened for LS were screened. 276 patients were evaluable. More minority women were screened as part of their routine cancer care (p = 0.005). Additionally, women 50 years or younger were more likely to be screened for LS compared to women older than 51(p = 0.009) and uninsured or reliant on Medicaid patients (p = 0.011) were more likely to be screened during routine care. Six patients received confirmatory germline testing for LS (4.3%), and another 8 patients had a staining pattern suggestive of LS. In an underrepresented population, the rate of LS in endometrial cancer is similar to previous reports. LS may be under diagnosed and opportunities missed when universal screening is not applied in minority women.

## Introduction

Lynch syndrome (LS) is an autosomal dominant disorder caused by germline inactivating mutations in DNA mismatch repair (MMR) genes, including *MSH2, MLH1, MSH6*, and *PMS2,* that lead to tumorigenesis. Women with LS have increased lifetime risks of both colon and endometrial cancer ranging between 40 and 60%, as well as a 25–50% risk of developing other cancers up to 15 years after initial diagnosis^1,2^. Strategies that allow for earlier diagnosis and cascade testing can prevent additional cancers and reduce cancer-related morbidity and mortality^3,4^.

Variation in LS screening strategies continue to exist despite known associations between LS and endometrial cancer. The Society of Gynecologic Oncology (SGO) suggests systematic clinical screening for LS in women diagnosed with endometrial cancer with review of personal and family history and/or molecular screening, with molecular screening as the preferred method if resources available. Germline testing for LS is recommended in women with endometrial or colorectal cancer with evidence of MSI or loss of MMR proteins, or in all women with a suggestive family history^5^. The American College of Obstetricians and Gynecologists (ACOG) recommends expanding the Bethesda Guidelines for LS screening in colon cancer patients, to include endometrial cancer as a sentinel cancer to identify women at risk of LS^6^. The National Comprehensive Cancer Network (NCCN) recommends universal screening for all patients with colorectal cancer, or if more limited screening is desired, those patients meeting the Bethesda Guidelines or Amsterdam Criteria^6^. However, the NCCN did not provide guidance for endometrial cancer screening until March 2019. LS screening relying on clinical judgment, age of diagnosis, and family history has been shown to fail at identifying up to 60% of all women with LS^7^.

Current data suggest that the incidence of LS among endometrial cancer cases is approximately 2–5%, largely in select high risk populations^7–10^. The largest published study which was mainly limited to patients of northern European decent, included 519 consecutive patients with endometrial cancer, identifying a germline MMR pathogenic variant rate of 2.1%^15^. Few studies to date have determined the prevalence of LS among minority women with endometrial cancer^11–16^. The primary objective of this study was to assess the prevalence of LS or likely LS in a minority patient population. Secondary objectives included identifying factors associated with primary LS screening and to characterize the clinicopathologic features in this cohort.

## Materials and methods

### Ethics statement

University of Miami Institutional Review Board approval was obtained (eprost #20170677). All experiments were performed in accordance with relevant guidelines and regulations. Patient informed consent was obtained as part of surgical consent at the time of surgery for IHC screening. Patients under the age of 18 years of age were not eligible. Patients were assigned study numbers and no individual data is included.

All patients treated for endometrial cancer at the University of Miami/Sylvester Comprehensive Cancer Center (CC) and Jackson Memorial Hospital, a large public safety net hospital (SNH), between January 2014 and December 2016 were evaluated for inclusion. Patients were excluded if they did not have surgery at our institution or if there was insufficient tumor tissue for analysis. Cohort 1 was comprised of women who had MMR IHC performed at initial surgical resection based on discretion of the treating physician as part of routine cancer care. Factors influencing physician decision include age at diagnosis, family history of cancers, consideration Bethesda or Amsterdam criteria or diagnosis of EC alone. Cohort 2 included all remaining patients who did not have initial MMR IHC screening at time of surgery with sufficient pathologic specimen remaining for retrospective IHC screening. For cohort 2, pathology specimens were reviewed by two pathologists (LG, AP) to confirm endometrial cancer diagnosis and to select optimal block for IHC. Thus, the combination of cohort 1 and 2 enables an analysis of MMR expression in unselected EC cases in a 3-year timespan. MMR panel IHC was performed using Roche’s antibody panel (MLH1 clone HMLH1, MSH2 clone G12-1129, MSH6 clone SP93, and PMS2 clone A16-4) in tumor tissue and considered abnormal if there was loss of protein expression in *MLH1, PMS2, MSH2*, and/or *MSH6*. The proportion of stained tumor epithelial component and intensity of staining was scored by two independent observers and tumor stroma was considered internal control. If MLH1/PMS2 or MLH1 IHC loss was detected, *MLH1* hypermethylation was assessed by polymerase chain reaction (PCR). Available LS genetic testing results were recorded. Genetic testing was performed as part of standard of care through commercially available platforms based on insurance coverage such as Ambry, Myriad, and Invitae. Women with abnormal MMR not due to *MLH 1* hypermethylation but without genetic testing results were classified as suspicious for LS. For those with abnormal MMR screening results, demographic data, parity, history of exogenous hormone use, tumor stage, tobacco use, insurance status, personal and family cancer history, IHC testing results, and genetic testing and/or referral to genetic counseling were abstracted from electronic medical records. Patients were assigned the following race/ethnicity categories based on self-identification at time of registration: Non-Hispanic White (NHW), Hispanic White (HW), Black (B), or Asian.

Statistical analyses were performed in R version 3.5.3 statistical software (only, figure and tables generated by authors). Chi square and Fisher’s exact tests were performed for bivariate categorical analyses. To assess differences in means, t-tests, and ANOVA were used with Tukey’s Honest Test for Significant Differences used post-hoc. To assess multivariate models, linear and logistic regression were used for categorical and continuous outcomes, respectively. All tests assumed a threshold of significance at alpha = 0.05.

## Results

**Figure 1:**
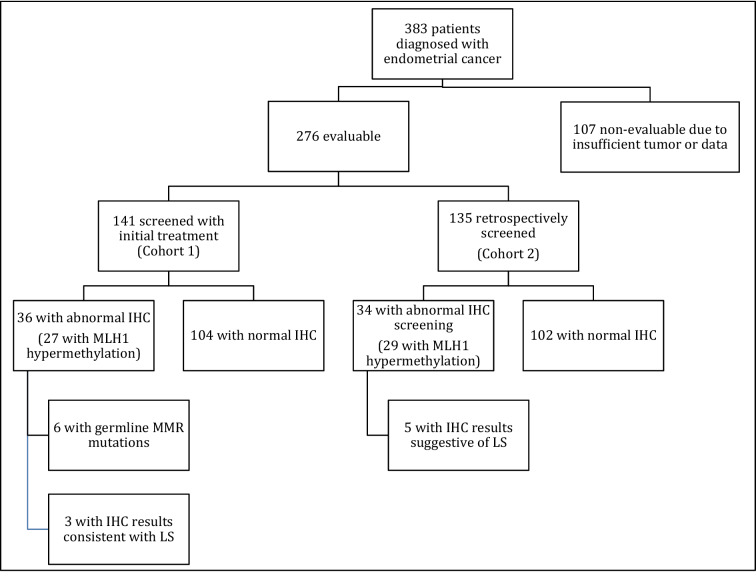
Study algorithm and screening results.

Between 2014 and 2016, 383 patients were diagnosed with endometrial cancer, of which 276 patients met inclusion criteria (Fig. 1).

Of the 276 patients, 141 (51.1%) had IHC screening as part of their routine cancer care (cohort 1), and 135 (48.9%) did not receive IHC screening at time of initial treatment and underwent subsequent testing (cohort 2). Stage (25, 9%), insurance (14, 5%), and smoking (10, 3.6%) data was not clearly documented for inclusion for data analysis. In both cohorts, the mean age at diagnosis was 60.1 years old (23–88), most had stage I disease (n = 166, 60.1%) and endometrioid histology (n = 206, 74.6%). Most patients (n = 219, 79.3%) were considered racial or ethnic minority (Table 1).

**Table 1:** Study demographics.

	Freq	%
**Sites**		
SNH	144	52.17
CC	132	47.83
Total	276	100
**Evaluation**		
Cohort 1 (initial)	141	51.09
Cohort 2 (retrospective)	135	48.91
Total	276	100
**IHC screening**		
Abnormal	70	25.36
Normal	206	74.64
Total	276	100
**Genetics referral**		
No	49	70
Yes	21	30
Total	70	100
**Genetic counseling/testing**		
No	58	82.86
Yes	12	17.14
Total	70	100
**LS diagnosis or suggestive of LS**		
Negative	56	80
Positive	14	20
Total	70	100
**Smoking**		
Nonsmoker	201	75.56
Smoker	65	24.44
Total	266	100
**Minority status**		
Minority	219	79.35
Non-minority	57	20.65
Total	276	100
**Race/ethnicity**		
Asian	2	0.72
HB	6	2.17
HW	147	53.26
NHB	64	23.19
NHW	57	20.65
Total	276	100
**Hispanic**		
H	153	55.43
NH	123	44.57
Total	276	100
**Age**		
≤ 50	42	15.22
51–60	103	37.32
61+	131	47.46
Total	276	100
**Insurance**		
Medicaid	49	18.7
Medicare	51	19.47
Private	84	32.06
Uninsured	78	29.77
Total	262	100
**Stage**		
1	166	66.14
2	13	5.18
3	39	15.54
4	33	13.15
Total	251	100
**Histology**		
Endometrioid	206	74.6
Serous	38	13.8
Carcinosarcoma	16	5.79
Clear cell	10	3.63
Mucinous	2	0.73
Mixed	4	1.45
**BMI**		
< 25	36	13.04
< 30	61	22.1
< 35	82	29.71
< 40	45	16.3
40+	52	18.84
Total	276	100

Women mostly identified as HW (53.3%) and B (25.4%) in our patient population.

Women treated at the SNH were more likely to have screening IHC performed as part of routine care (p < 0.001). More racial and ethnically under-represented patients were screened in cohort 1 than in cohort 2 (124 in cohort 1 vs 98 in cohort 2) (p = 0.004) (Table 2).

**Table 2:** Demographic characteristics of patients originally screened as part of routine cancer care and those retrospectively screened as part of this study.

	Cohort 1 (initial) (n = 141)	Cohort 2 (retrospective) (n = 135)	p-value
**Site**			
SNH	125 (86.81%)	19 (13.19%)	
CC	16 (12.12%)	116 (87.88%)	
Col total	141 (51.09%)	135 (48.91%)	
**IHC screening**			
Abnormal	36 (51.43%)	34 (48.57%)	
Normal	105 (50.97%)	101 (49.03%)	
**Genetic counseling/testing**			
No	24 (41.38%)	34 (58.62%)	
Yes	12 (100%)	0 (0%)	0.001
**LS diagnosis or suggestive of LS**			
Negative	27 (48.21%)	29 (51.79%)	
Positive	9 (64.29%)	5 (35.71%)	0.437
**Smoking**			
Nonsmoker	98 (48.76%)	103 (51.24%)	
Smoker	35 (53.85%)	30 (46.15%)	0.568
**Minority status**			
Minority	122 (55.71%)	97 (44.29%)	
Non-minority	19 (33.33%)	38 (66.67%)	0.004
**Race**			
Asian	0 (0)%	2 (100%)	
Black	37 (52.86%)	33 (47.14%)	
White	104 (50.98%)	100 (49.02%)	0.47
**Race/ethnicity**			
Asian	0 (0%)	2 (100%)	
HB	5 (83.33%)	1 (16.67%)	
HW	85 (57.82%)	62 (41.18%)	
NHB	32 (50%)	32 (50%)	
NHW	19 (33.33%)	38 (66.67%)	0.004
**Hispanic**			
H	90 (58.82%)	63 (41.18%)	
NH	51 (41.46%)	72 (58.54%)	0.006
**Age**			
≤ 50	29 (69.05%)	13 (30.95%)	
51–60	56 (54.37%)	47 (45.63%)	
61+	56 (42.75%)	75 (57.25%)	0.009
**Insurance**			
Medicaid	33 (67.35%)	16 (32.65%)	
Medicare	19 (37.25%)	32 (62.75%)	
Private	40 (47.62%)	44 (52.38%)	
Uninsured	46 (58.97%)	32 (41.03%)	0.011
**Stage**			
I	84 (50.6%)	82 (49.4%)	
II	8 (61.54%)	5 (38.46%)	
III	25 (64.1%)	14 (35.9%)	
IV	14 (42.42%)	19 (57.58%)	0.251
**BMI**			
< 25	16 (44.44%)	20 (55.56%)	
< 30	28 (45.9%)	33 (54.1%)	
< 35	44 (53.66%)	38 (46.34%)	
< 40	28 (62.22%)	17 (37.78%)	
40+	25 (48.08%)	27 (51.92%)	0.415

Specifically, Hispanic women were more likely to be screened (p = 0.006) but race did not affect screening (p = 0.47). The median age was younger among women in cohort 1 compared to cohort 2 (58 years old in cohort 1 vs. 61 years old in cohort 2) (p = 0.005). Women receiving genetic counseling were younger compared to women who did not (median age 54 vs 61.5, p = 0.007). Women 50 years or younger were more likely to be screened for LS compared to women older than 60 years (p = 0.009). Additionally, women greater than 61 years old had a lower likelihood of initial screening but there was no significant difference in the likelihood of initial screening in women younger than 50 compared to women 51–60 years old (OR 2.23, 95% CI 1.16–4.29, p = 0.01). Those who were uninsured or reliant on Medicaid were more likely to be screened at initial treatment compared to those with Medicare or have private insurance (p = 0.011) (Table 3).

**Table 3:** Demographic characteristics of patients with normal and abnormal MMR screening. No categories predicted abnormal screening results.

	Abnormal MMR (n = 70)	Normal MMR (n = 206)	p-value
**Minority status**			
Minority	61 (27.85%)	158 (72.15%)	
Non-minority	9 (15.79%)	48 (84.21%)	0.09
**Race/ethnicity**			
Asian	1 (50%)	1 (50%)	
HB	1 (16.67%)	5 (83.33%)	
HW	40 (27.21%)	107 (72.79%)	
NHB	19 (29.69%)	45 (70.31%)	
NHW	9 (15.79%)	48 (84.21%)	0.261
**Hispanic**			
H	41 (26.8%)	112 (73.2%)	
NH	29 (23.58%)	94 (76.42%)	0.637
**Age**			
≤ 50	9 (21.43%)	33 (78.57%)	
51–60	26 (25.24%)	77 (74.76%)	
61+	35 (26.72%)	96 (73.28%)	0.79
**Insurance**			
Medicaid	15 (30.61%)	34 (69.39%)	
Medicare	14 (27.45%)	37 (72.55%)	
Private	24 (28.57%)	60 (71.43%)	
Uninsured	13 (16.67%)	65 (83.33%)	0.22
**Stage**			
I	43 (25.9%)	123 (74.1%)	
II	3 (23.08%)	10 (76.92%)	
III	8 (20.51%)	31 (79.49%)	
IV	12 (36.36%)	21 (63.64%)	0.479
**BMI**			
< 25	9 (25%)	27 (75%)	
< 30	19 (31.15%)	42 (68.85%)	
< 35	20 (24.39%)	62 (75.61%)	
< 40	9 (20%)	36 (80%)	
40+	13 (25%)	39 (75%)	0.771

There was no difference in mean BMI between cohort 1 (33.22 kg/m^2^) and cohort 2 (34.07 kg/m^2^) (p = 0.386). When comparing those with abnormal IHC screening to those with normal screening, no significant differences were noted across race or ethnicity (p = 0.261), minority status (p = 0.09), age (p = 0.79), insurance status (p = 0.22), stage (p = 0.479), or BMI (0.771) (Table 3). Furthermore, adjusted odd ratios for significant bivariate effects associated with initial screening were only significant for patients receiving care at SNH compared to CC (aOR 10.27 95% CI 2.6–40.5; p = 0.009) and patients with private insurance were less likely to be screened in cohort 1 (aOR 11.88 95% 2.07–68.1; p = 0.005).

A total of seventy (25.1%) patients from both cohorts had abnormal MMR IHC screening. In cohort 1, nine women were either diagnosed with LS based on genetic testing or had IHC results suggestive of LS with an additional five women from cohort 2 demonstrating IHC suggestive of LS. Six women from cohort 1 completed genetic counseling and testing confirmed diagnosis of LS (4.3% of a selected EC population). Fourteen (5%) women in the total cohort were diagnosed with LS or MMR IHC suspicious for LS. Of the patients diagnosed with or had MMR loss, twelve (85.7%) women were racial and ethnically underserved; median BMI was 28.7 (22.9–44.3); four previously used exogenous estrogen therapy (28.5%); two (14%) had a prior LS-associated malignancy; and five (35.7%) had a family history significant for known LS-associated malignancies (Table 4).

**Table 4:** Demographics and risk factors of suspected or diagnosed LS patients.

Cohort	IHC loss	Genetic testing	Age Dx	Minority status	BMI	Personal cancer Hx	Prior hormone	Family cancer history	Final Dx
1	PMS2	PMS 2 p.M1V	57	No	43.1	No	OCPs then IUD	Maternal aunt: breast ca age 67 Maternal grandmother: stomach ca age 84 Maternal grandfather: pancreatic ca age 86	Yes
1	MLH1PMS2	MLH1 c.193_201delGGCACCGGGinsC	40	Yes	23.5	No	Unknown	Paternal uncle: colon ca age 50	Yes
1	MLH1 PMS2	MLH 1 c.380 + 2 T > A	51	No	30.2	Myxofibrosarcoma CRC	OCPs then IUD	Mother: endometrial ca age 41 Brother: colon ca age 44 Paternal aunt: breast ca in 50 s	Yes
1	PMS2	PMS2 c.690_691delGT	61	Yes	29.3	No	No	No	Yes
1	MSH6	MSH6 (c.3849_3851delTACins10)	54	Yes	40.1	No	No	Mother: endometrial ca Sister: breast ca Unknown ages	Yes
1	MSH2 MSH6	MSH2 c 1351C > T (pGln451)	52	Yes	28.9	No	No	No	Yes
1	PMS2	P^a^	58	Yes	36.5	No	No	Mother: endometrial ca Father colon ca Unknown ages	
1	PMS2	P^a^	58	Yes	36.0	No	No	No	
1	MSH2MSH6	P^b^	62	Yes	27.3	No	No	No	
2	MSH6	P^b^	60	Yes	24.5	No	No	No	
2	MSH2MSH6	P^b^	59	Yes	26.6	No	Vaginal estrogen	No	
2	PMS2	P^b^	63	Yes	44.3	No	No	No	
2	PMS2	P^d^	56	Yes	22.9	No	OCPs then HRT	No	
2	MSH2	P^c^	65	Yes	23.8	No	No	No	

^a^Referred to genetic counselor, then lost to follow up.

^b^Referred to genetic counselor (did not go).

^c^Lost to follow up.

^d^No genetic testing performed to date.

Most patients (n = 53, 75%), with abnormal screening results had loss of both MLH1 and PMS2 proteins. Thirty-nine (73.6%) of these had *MLH 1* promoter hypermethylation in subsequent testing but 11 (20.8%) specimens had insufficient tissue for hypermethylation testing. Isolated loss of *PMS2* occurred in six (8.5%) patients. Concurrent loss of *MSH2* and *MSH6* occurred in five (7%) patients. Isolated loss of *MSH6* occurred in three (4.2%) patients; isolated loss of *MSH2* in two (2.6%). We identified the following mutations in *PMS2,* p.M1V, c.193_201delGGCACCGGGinsC, c.380 + 2T > A, *PMS2* c.690_691delGT; in *MSH6*, c.3849_3851delTACins10; and in *MSH2*, c 1351C > T (pGln451).

## Discussion

ACOG and SGO have proposed following one of three approaches to screening for LS among women with endometrial cancer^17^. The approaches include (1) LS tumor testing on any woman found to be at risk for LS by personal or family history; (2) performing tumor testing on all endometrial cancers diagnosed before age 60; or (3) performing tumor testing on all endometrial tumors regardless of age of diagnosis. Similarly, NCCN guidelines are multi-tiered and complex but more recently, recommended MMR screening for all endometrial cancer patients regardless of age^6^. Not surprisingly, considerable variability remains among endometrial cancer patients being screened for LS. In a study by Hempel et al., including 543 endometrial cancer patients, 10 patients were identified with LS, of which 7 did not meet published criteria for LS screening. Thus, the authors proposed screening all patients diagnosed with endometrial cancer for LS^7^. This study was initiated in 2016, however, due to logistical challenges (cases were retrospectively identified, pathologic specimens retrieved and reviewed, review of medical records to abstract clinical information and change in personnel), the manuscript was not ready for submission until 2019. In cohort 1, of select EC cases, LS was diagnosed in 4.3% and another 3 (33%) with IHC suggestive of LS, who were referred for genetic counseling and testing but did not go to appointment. Across both cohorts of 14 patients with diagnosed or suspected LS, 9 would have met screening recommendations based on an age < 60, 5 would have met screening guidelines based on a positive family history suggestive of LS, and 5 patients met neither criteria. Identifying a pathogenic variant in a family presents a critical opportunity to perform enhanced screening for other LS related malignancies and to provide predictive testing for at-risk family members.

Prior studies identifying the frequency of LS among patients with endometrial cancer report a prevalence of 2–6%^18–20^. However, these studies consisted of either a majority European or NHW population, or did not detail the racial and ethnic makeup of their study populations^11,15,16,21^. Reported founder mutations for LS have been identified in Iceland, Finland, the Netherlands, and Sweden, promoting a belief that LS primarily occurs among individuals of Northern European descent^22–25^. To date studies describing the prevalence and type of mutations among minority patient populations in the US are lacking. In a study among patients with colorectal cancer, lower rates of genetic counseling referrals in minority patients with MMR tumor mutations were described^26^. In one of the first studies to report on the prevalence of LS among Hispanic patients diagnosed with endometrial cancer, 15.7% of tumors demonstrated MMR deficiency (9 MSH6, 3 MSH2, and 1 MLH1) suggestive of LS^27^. This study suggests that risk of LS among Hispanic patients is at least as high as the prevalence in other populations. In another study, Lee et al. evaluated genetic counseling referrals and testing in an ethnically diverse group of high-risk women diagnosed with endometrial cancer. In their cohort, 58% of women were White while minority populations consisted of 13.6% Black, 14.6% Latina, and 9.9% Asian, and only approximately 40% had MMR screening performed. LS was identified in 1.7% of the entire study cohort, however only 35% of patients were tested^28^. Our study, comprised largely of racial and ethnically underserved women (80%), had a prevalence of LS-related endometrial cancer consistent with that in other populations, further supporting need for awareness and for screening in this population to close the gap in health disparities. Recognizing women with MMR loss identifies novel therapeutic options (checkpoint inhibitor)^29^ as well as opportunities for cancer prevention in LS carriers.

Genetic testing in gynecologic cancers has historically been low due to barriers in delivery of genetic testing^30–33^. Barriers to testing requiring intervention were identified and categorized as provider-mediated, payer-associated, system-associated, and patient-associated factors in a 2017 SGO white paper^34^. At our institution, we recognized that despite national guidelines, approximately 50% of women diagnosed with endometrial cancer were not routinely being screened. Thus, as part of a quality improvement process, in collaboration with pathology colleagues, universal MMR testing was implemented on 5/1/2016. In our study, more minority women, uninsured or Medicaid patients were screened in cohort 1 reflecting care provided at our safety net hospital, suggesting that provider-mediated barriers are not insurmountable. This also highlights the importance of provider education and reinforcement of societal recommendations as well as optimizing the operational process in the first step of LS screening. When LS is diagnosed, it allows for appropriate screening to prevent other cancers within the same individual as well as cascade testing for family members^35^. Cascade testing of unaffected relatives offers the largest advantage in terms of life-years saved from primary cancer prevention^35^.

While this study demonstrates important data trends that may contribute to clinical decision-making in patients with endometrial cancer, certain limitations exist. The study is limited by its retrospective nature as well as selection bias due to exclusion of patients with lack of remaining tumor for additional testing. Certainly, the capability to perform IHC testing for all patients retrospectively as part of cohort 2 would have strengthened the results (exclusion of 107 patients with insufficient tissue). Additionally, following completion of retrospective screening (cohort 2), women for which germline testing would be indicated had been lost to follow up. The strengths of this study are that to the best of our knowledge, this study is the first to report on the prevalence of LS in a primarily underserved population diagnosed with endometrial cancer. Furthermore, we demonstrated the ability to perform universal MMR testing, which is relatively inexpensive, even at a safety net hospital system where resources are often limited.

The results of our study demonstrate similar rates of LS among minority women diagnosed with endometrial cancer as those reported in larger NHW patient populations. Our data is consistent with published literature demonstrating a cohort of LS would have been missed had universal tumor screening not occurred. Furthermore, racial, and ethnically underserved populations have LS at frequencies similar to non-minority populations and adherence to guidelines should be emphasized.
